# Subregions of the Anterior Cingulate Cortex Form Distinct Functional Connectivity Patterns in Young Males With Internet Gaming Disorder With Comorbid Depression

**DOI:** 10.3389/fpsyt.2018.00380

**Published:** 2018-08-29

**Authors:** Deokjong Lee, Junghan Lee, Kee Namkoong, Young-Chul Jung

**Affiliations:** ^1^Psychiatry, National Health Insurance Service Ilsan Hospital, Goyang-si, South Korea; ^2^Institute of Behavioral Science in Medicine, College of Medicine, Yonsei University, Seoul, South Korea; ^3^Psychiatry, College of Medicine, Yonsei University, Seoul, South Korea

**Keywords:** anterior cingulate cortex, default mode network, depression, functional connectivity, Internet Gaming Disorder

## Abstract

Depression is one of the most common comorbid conditions in Internet Gaming Disorder (IGD). Although there have been many studies on the pathophysiology of IGD, the neurobiological basis underlying the close association between depression and IGD has not been fully clarified. Previous neuroimaging studies have demonstrated functional and structural abnormalities in the anterior cingulate cortex (ACC) in IGD patients. In this study, we explored functional connectivity (FC) abnormalities involving subregions of the ACC in IGD subjects with comorbid depression. We performed a resting state seed-based FC analysis of 21 male young adults with IGD with comorbid depression (IGDdep+ group, 23.6 ± 2.4 years), 22 male young adults without IGD with comorbid depression (IGDdep− group, 24.0 ± 1.6 years), and 20 male age-matched healthy controls (24.0 ± 2.2 years). ACC-seeded FC was evaluated using the CONN-fMRI FC toolbox. The dorsal ACC (dACC), the pregenual ACC (pgACC), and the subgenual ACC (sgACC) were selected as seed regions. Both IGD groups had stronger pgACC FC with the right precuneus, the posterior cingulate cortex, and the left inferior frontal gyrus/insula than the control group. The IGDdep+ group had stronger dACC FC with the left precuneus and the right cerebellar lobule IX than the control and IGDdep- groups. The IGDdep+ group also had weaker pgACC FC with the right dorsomedial prefrontal cortex and the right supplementary motor area and had weaker sgACC FC with the left precuneus, the left lingual gyrus, and the left postcentral gyrus than the other groups. The strength of the connectivity between the sgACC and the left precuneus correlated positively with a higher omission error rate in the continuous performance test in the IGDdep+ group. In addition, the IGDdep– group had stronger sgACC FC with the left dorsolateral prefrontal cortex than the other groups. Our findings suggest that young males with IGD comorbid with depression have FC alterations of the default mode network and diminished FC with the prefrontal cortex. This altered FC pattern may be involved in the close association of IGD and depression.

## Introduction

During the past decade, much research has been conducted on Internet Gaming Disorder (IGD), which is characterized by a difficulty in controlling Internet game use despite psychosocial disturbance (1). The high rate of comorbidity and the causal relationship between IGD and other psychiatric diseases have attracted much attention (2). Depression is a common comorbid psychiatric condition in IGD, and the comorbidity of IGD and depression has been related to more serious psychosocial burdens (3). A maladaptive emotional regulation strategy that suppresses rather than uses cognitive reappraisal of emotion has been presented as a contributing factor to the comorbidity of IGD and depression (4). Several neurobiological factors, such as decreased inter-hemispheric connectivity of the frontal regions and structural alterations in the dorsolateral prefrontal cortex, have been suggested to mediate the relationship between IGD and depressed mood (5, 6). Although these previous studies have improved our understanding of the associations between IGD and depression, research on the relationship between IGD and depression remains scarce despite its high clinical significance. Because a consensus on therapeutic tools for IGD is still lacking (7), further understanding of the associations between IGD and depression could provide new targets for IGD intervention. For instance, a recent study reported that bupropion was more effective than escitalopram as a treatment for IGD patients with comorbid depression (8).

Evidence has indicated that structural and functional dysfunctions of the anterior cingulate cortex (ACC) underlie the development and maintenance of IGD (9). Altered interactions between the ACC and other regions of the brain may contribute to the development of IGD and its related clinical characteristics. The linkages between the ACC and other regions of the brain are complex; each of the subregions of the ACC connect to different regions of the brain with different and specific functions (10). It has been suggested that the dorsal ACC (dACC) is involved in attentional and executive control via connections with the dorsolateral prefrontal cortex (DLPFC) (11, 12) and that the rostral ACC (rACC) is involved in emotional processing via connections with the amygdala, hippocampus, and the orbitofrontal cortex (OFC) (13). The rACC is divided into the pregenual ACC (pgACC) and the subgenual ACC (sgACC) (14). The pgACC has been shown to have dense connectivity with the lateral prefrontal cortex and plays an important role in top-down regulation of emotional stimuli (15). The sgACC has been found to have strong connectivity with the amygdala and the ventral striatum and contributes to autonomic control and conditioning learning for emotional processing (16).

Resting state functional connectivity (FC) between the ACC and other regions of the brain can be used to evaluate the interactions of the ACC with the other regions of the brain. Previous resting state functional magnetic resonance imaging (fMRI) studies showed that individuals with IGD had reduced FC between the dACC and some of the subcortical regions of the brain, including the dorsal striatum, the pallidum, and the thalamus, and increased FC between the rACC and the anterior insula (17, 18). These findings are consistent with the view that diminished executive control and enhanced reward seeking may underlie IGD (19). In IGD patients with comorbid depression, comorbidity with depression associated with reduced suppression of the default mode network (DMN), which may contribute to the attentional problems (20). The DMN and its interactions with other brain networks were found to play important roles in depression (21). It has been suggested that the DMN during the depressed state includes the rACC, especially the sgACC (22, 23). Individuals with depression have been shown to have increased FC between the sgACC and areas of the anterior DMN (24) and the salience network (SN) (25). Thus, both IGD and depression alter the FC of the subregions of the ACC. These FC alterations may contribute to the comorbidity of IGD and depression and its related clinical characteristics, but more research is needed on the relationships between IGD and depression and FC alterations.

The executive function is the higher order cognitive processes that is essential for proper control over behavior, and previous studies have demonstrated that executive functions are impaired in IGD (26), for instance, subjects with IGD showed high impulsivity, which is an example of diminished executive control (27, 28). Executive deficits have also been associated with depression (29), for example, depressed patients have demonstrated altered attentional control (30), thus attentional control has been a therapeutic target for depression (31). Executive deficit is an important component of the pathophysiology and clinical manifestations of IGD and depression. However, the exact role of the executive function in the relationship between IGD and depression has not yet been fully elucidated.

The aim of this study was to investigate the ACC-seeded FC of IGD subjects with depression. Three subregions of the ACC, the dACC, the pgACC, and the sgACC, were analyzed. We hypothesized that IGD subjects would show different patterns of ACC-based FC depending on whether comorbid depression was present or not. Based on previous studies, we expected that subjects with IGD would have reduced FC between the dACC and the subcortical regions and increased FC between the rACC (pgACC or sgACC) and seeds of the SN regardless of the presence of comorbidity with depression. We also expected that FC between the sgACC and other DMN- or SN-related seed regions would be higher in IGD subjects with comorbid depression reflecting their DMN abnormalities. We tested these expectations through resting state seed-based FC analysis, and we examined correlations between FC alterations and executive functions in IGD patients with comorbid depression. Impulsivity and attentional processes, which are clinical variables of executive functions, were assessed with self-reporting questionnaires for impulsivity and a continuous performance test (CPT) for attentional processes.

## Methods

### Subjects

This study was conducted from February 2015–April 2017, and the protocols for this study were approved by the Institutional Review Board at Severance Hospital, Yonsei University. Subjects were recruited via online advertisements, flyers, and word of mouth. All of the subjects were informed of the entire procedure and signed an informed consent before participating in the study.

We screened 101 young male adults for this study. According to previous epidemiological studies, IGD is more common in males (32). Because there are gender differences in the behavioral characteristics and motives for online gaming (33), this study was conducted only for men to reduce confounding effect. Subjects were examined for their Internet usage patterns and they completed Young's Internet Addiction Test (IAT) (34). Subjects who used the Internet primarily for gaming and whose IAT scores (34) exceeded 50 were interviewed according to the IGD diagnostic criteria of the DSM Fifth Edition to determine whether IGD was present (35). Subsequently, subjects with IGD were assessed for depression using the Beck Depression Inventory (BDI) (36). Among the subjects with IGD, those with a BDI score of 20 or higher were classified as IGD subjects with comorbid depression, whereas those with a BDI score of 13 or lower were classified as IGD subjects without comorbid depression. All of the subjects were assessed for their intelligence quotient (IQ) using the Wechsler Adult Intelligence Scale-Fourth Edition (WAIS-IV) (37). All of the subjects were also assessed for the presence of major psychiatric disorders using the Structured Clinical Interview from the DSM Fourth Edition (SCID-IV) (38). All subjects with a BDI score of 20 or higher were confirmed to have current depression (satisfying the criteria of mild depressive episode or major depressive episode). Subjects with the following were excluded: a neurological disorder or medical illness, major psychiatric illness other than IGD or depression (i.e., bipolar disorder, psychotic disorder, substance use disorder, attention deficit/hyperactivity disorder), mental retardation, or radiological contra-indications on the MRI scan.

After the screening process, 63 young male adults 20–27 years of age (mean: 23.8 ± 2.0 years) participated in the study, and all of them were right-handed. Subjects with IGD were subdivided into two groups according to their comorbid depression: IGD subjects with comorbid depression (IGDdep+ group, *n* = 21; 23.6 ± 2.4 years) and IGD subjects without comorbid depression (IGDdep- group, *n* = 22; 24.0 ± 1.6 years). Subjects who spent less than 2 h per day on gaming and scored below 50 points on the IAT were classified as healthy controls (*n* = 20; 24.0 ± 2.2 years). In addition to the IAT and BDI used in the screening process, subjects completed the Alcohol Use Disorders Identification Test (AUDIT) (39), the Beck Anxiety Inventory (BAI) (40), and the Barratt Impulsiveness Scale-version 11 (BIS-11) self-reporting questionnaires (41).

### Continuous performance test (CPT)

We applied the computerized Comprehensive Attention Test to assess the abilities of sustained attention and divided attention (42). In the sustained attention task, various shapes are presented on the computer screen every 2 s as a visual stimulus, and the task is performed for 10 min. Subjects were instructed to press the space bar as quickly as possible whenever visual stimuli were displayed, but not when an “X” shape was presented. The sustained attention task assesses the ability to exert consistent behavioral responses while sustaining attention to continuous and repetitive stimuli. This task also estimates impulsivity by assessing whether a subject could suppress behavioral responses to specific stimuli. In the divided attention task, visual and auditory stimuli are presented at the same time every 2 s, and the task takes a total of 3 min and 20 s. Subjects were instructed to press the spacebar as quickly as possible in the event that the immediately preceding visual stimulus or auditory stimulus is presented again. The divided attention task assesses whether subjects can process two or more stimuli simultaneously by properly dividing their attention. Two behavioral variables were measured for performance on the CPT. The omission error is the failure to perform a required behavioral response and it reflects inattention. The commission error is the presence of behavioral responses that should have been suppressed and it reflects impulsivity.

### MRI image acquisition and pre-processing

MRI images were acquired using a 3T Siemens Magnetom MRI scanner equipped with an eight-channel head coil. The fMRI data were collected using a single-shot T2-weighted gradient echo planar pulse sequence (echo time = 30 ms, repetition time = 2,200 ms, flip angle = 90°, field of view = 240 mm, matrix = 64 × 64, slice thickness = 4 mm) for 6 min. Subjects were instructed to gaze at the white crosshair in the center of the black background without any cognitive, lingual, or motor activity. An anatomical template for the fMRI data was acquired using a T1-weighted spoiled gradient echo sequence (TE = 2.19 ms, TR = 1,780 ms, flip angle = 9°, field of view = 256 mm, matrix = 256 × 256, slice thickness = 1 mm). Pre-processing and statistical analysis of the data were performed using SPM8 (Welcome Trust Centre for Neuroimaging; http://www.fil.ion.ucl.ac.uk/spm). For each subject, the initial seven points in the time series were discarded to eliminate signal decay. To adjust for motor artifacts for each subject, we checked that the maximal head movement in each axis was < 2 mm and that there was no unexpected head motion by visually inspecting realignment parameter estimates. For each subject, functional brain images were realigned and co-registered to structural images. The co-registered images were spatially normalized to the Montreal Neurological Institute (MNI) template (provided by SPM8) using a 12-parameter affine transformation and non-linear iterations. Parameters of normalization were applied to unwrapped functional images, which were then re-sampled to a voxel size of 2 × 2 × 2 mm. Data were smoothed using an 8 mm full-width at half-maximum kernel.

### FC analysis

Seed-to-voxel FC maps for each subject were constructed using the CONN-fMRI FC toolbox (http://www.nitrc.org/projects/conn). Seed regions for subregions of the ACC were defined as 5 mm radius sphere-centered coordinates derived from previous FC studies (dACC: 4 14 36; pgACC: −2 44 20; sgACC: 2 20−10) (43, 44). The waveform of each brain voxel was temporally filtered by means of a bandpass filter (0.008 Hz < f < 0.09 Hz) to adjust for low-frequency drift and high-frequency noise effects. A linear regression analysis was conducted to remove signals from the ventricular area and the white matter (45). To minimize the effects of head movement, motion parameters were entered into the linear regression analysis. To estimate the strength of an FC, correlation coefficients were computed and converted to z-values using Fisher's r-to-z transformation. Then, FC strength estimates were compared between groups using analysis of variance (ANOVA) at each voxel. As statistical inferences for the exploratory whole-brain analysis, a cluster forming threshold using a height threshold of uncorrected *p*-value < 0.001 and an extent threshold of 100 contiguous voxels was applied. After clusters with significant group differences were evaluated, Bonferroni *post hoc* tests were performed to examine which groups were different from the others.

### Statistical analysis

One-way ANOVA tests were used to compare demographic and clinical variables, including age, IQ, IAT, AUDIT, BDI, BAI, and BIS scores, among the three groups. Because the assumptions for normality were not met, comparisons of behavioral performance on the CPT between the groups were analyzed using the Kruskal Wallis test. The Bonferroni correction was applied for *post hoc* analysis. Partial correlation analysis of connectivity strength, BIS subscales, and behavioral performance of the CPT was performed after controlling for BDI and BAI. Statistical analyses were performed with SPSS (Chicago, IL) with significance set at *p* < 0.05 (two-tailed).

## Results

### Demographic and clinical variables of subjects

Controls and IGD subjects did not differ significantly in age, IQ, and AUDIT score (Table 1). Psychometric self-report scales showed differences in IAT [*F*_(2, 60)_ = 111.949, *p* < 0.001], BDI [*F*_(2, 60)_ = 185.146, *p* < 0.001], and BAI [*F*_(2, 60)_ = 30.498, *p* < 0.001] scores. BIS subscales differed between groups [non-planning: *F*_(2, 60)_ = 11.229, *p* < 0.001; motor: *F*_(2, 60)_ = 11.246, *p* < 0.001; cognitive: *F*_(2, 60)_ = 11.019, *p* < 0.001]. *Post hoc* testing showed that both IGD groups had significantly higher IAT and BIS scores than the control group. The IGDdep+ group showed higher BDI and BAI scores than the other groups. Comparison of behavioral performance on the CPT showed differences only in the omission error rate in the divided attention task (χ ^2^ = 6.130, *p* = 0.047). *Post hoc* testing showed that the IGDdep+ group had a higher omission error rate than the other groups.

**Table 1:** Demographic and clinical variables of subjects.

	**Controls (*n* = 20)**	**IGD_dep−_(*n* = 22)**	**IGD_dep+_ (*n* = 21)**	**Test**	***p*-value**	***Post hoc* test**
Age, year	24.0 ± 2.2	24.0 ± 1.6	23.6 ± 2.4	*F*_(2, 60)_ = 0.267	0.767	
Full Scale IQ	107.9 ± 10.7	109.9 ± 11.9	102.2 ± 12.5	*F*_(2, 60)_ = 2.452	0.095	
IAT	26.4 ± 9.8	69.4 ± 12.5	71.7 ± 10.1	*F*_(2, 60)_ = 111.949	< 0.001	IGD_dep−_, IGD_dep+_ > HC
BDI	5.0 ± 3.5	7.6 ± 3.4	25.6 ± 4.3	*F*_(2, 60)_ = 185.146	< 0.001	IGDdep+ > HC, IGDdep-
BAI	4.8 ± 4.4	6.7 ± 5.1	19.9 ± 9.7	*F*_(2, 60)_ = 30.498	< 0.001	IGDdep+ > HC, IGDdep-
AUDIT	9.8 ± 7.1	14.1 ± 7.5	11.5 ± 7.8	*F*_(2, 60)_ = 1.768	0.179	
**BIS SCALES**						
Non-planning impulsivity	16.5 ± 5.6	25.6 ± 7.7	22.9 ± 5.4	*F*_(2, 60)_ = 11.229	< 0.001	IGD_dep−_, IGD_dep+_ > HC
Motor impulsivity	12.9 ± 3.3	18.5 ± 4.4	17.7 ± 4.4	*F*_(2, 60)_ = 11.246	< 0.001	IGD_dep−_, IGD_dep+_ > HC
Cognitive impulsivity	11.2 ± 4.0	15.0 ± 2.7	16.1 ± 3.7	*F*_(2, 60)_ = 11.019	< 0.001	IGD_dep−_, IGD_dep+_ > HC
**SUSTAINED ATTENTION TASK, NUMBER**						
Omission error	1.4 ± 2.6	1.1 ± 1.6	1.6 ± 3.6	χ^2^ = 0.114	0.944	
Commission error	5.4 ± 3.0	8.3 ± 7.0	9.2 ± 9.2	χ^2^ = 1.163	0.559	
**DIVIDED ATTENTION TASK, NUMBER**						
Omission error	4.7 ± 6.1	5.4 ± 8.1	10.3 ± 10.4	χ^2^ = 6.130	0.047	IGDdep+ > HC, IGDdep-
Commission error	3.5 ± 2.2	3.4 ± 5.2	4.3 ± 7.8	χ^2^ = 1.786	0.409	

*Group comparisons were conducted by one-way analysis of variance (ANOVA) tests. Because assumptions for normality were not met for the behavioral variables for the attention tasks, the Kruskal Wallis test was used for comparison*.

*IGD_dep−_, Internet Gaming Disorder subjects without comorbid depression; IGD_dep+_, Internet Gaming Disorder subjects with comorbid depression; IQ, intelligence quotient; IAT, Internet Addiction Test; BAI, Beck Anxiety Inventory; BDI, Beck Depression Inventory; AUDIT, Alcohol Use Disorders Identification Test; BIS, Barratt Impulsiveness Scale*.

### FC analysis

**Figure 1:**
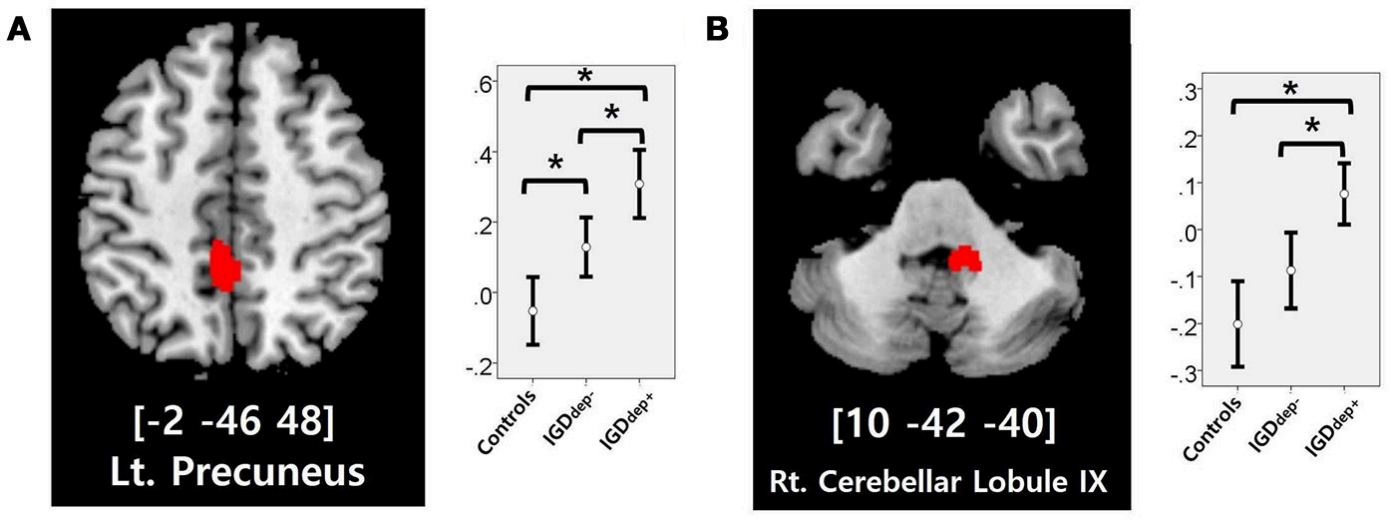
Brain regions showing significant differences in dACC-based FC between groups. **(A)** Left precuneus and **(B)** right cerebellar lobule IX. Height threshold of uncorrected *p*-value < 0.001 and extent threshold of 100 contiguous voxels. The peak coordinates of each cluster are indicated by the Montreal Neurological Institute (MNI) system. *Post hoc* tests were conducted to detect differences across groups using the Bonferroni correction. *p < 0.05.

**Figure 2:**
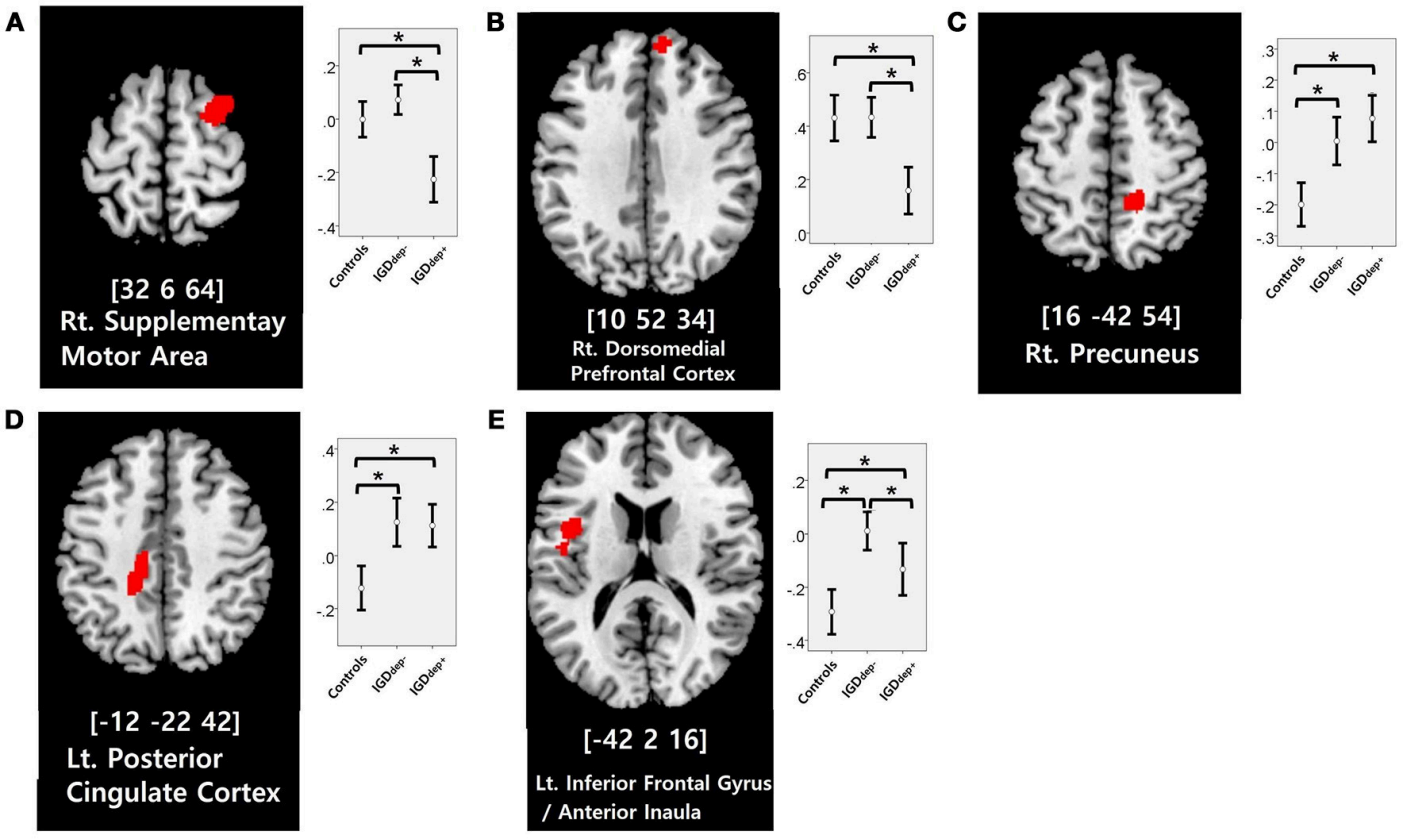
Brain regions showing significant differences in pgACC-based FC between groups. **(A)** Right supplementary motor area, **(B)** right dorsomedial prefrontal cortex, **(C)** right precuneus, **(D)** left posterior cingulate cortex, and **(E)** left inferior frontal gyrus/anterior insula. Height threshold of uncorrected *p*-value < 0.001 and extent threshold of 100 contiguous voxels. The peak coordinates of each cluster are indicated by the Montreal Neurological Institute (MNI) system. *Post hoc* tests were conducted to detect differences across groups using the Bonferroni correction. *p < 0.05.

**Figure 3:**
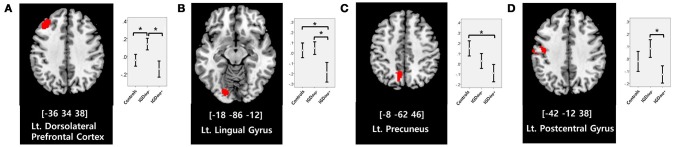
Brain regions showing significant differences in sgACC-based FC between groups. **(A)** Left dorsolateral prefrontal cortex, **(B)** left lingual gyrus, **(C)** left precuneus, and **(D)** left postcentral gyrus. Height threshold of uncorrected *p*-value < 0.001 and extent threshold of 100 contiguous voxels. The peak coordinates of each cluster are indicated by the Montreal Neurological Institute (MNI) system. *Post hoc* tests were conducted to detect differences across groups using the Bonferroni correction. *p < 0.05.

In the whole-brain analysis, multiple clusters with significant differences in FC were found between the groups (Table 2). The dACC-based FC analysis showed that the IGDdep+ group had stronger dACC FC with the left precuneus and the right cerebellar lobule IX than the other groups (Figure 1). The pgACC-based FC analysis showed that the IGDdep+ group had weaker pgACC FC with the right dorsomedial prefrontal cortex (dmPFC) and the right supplementary motor area (SMA) than the other groups (Figure 2). Both IGD groups had stronger pgACC FC with the right precuneus, the left posterior cingulate cortex (PCC), and the left inferior frontal gyrus/anterior insula (IFG/AI) than the controls. The sgACC-based FC analysis showed that the IGDdep+ group had weaker sgACC FC with the left precuneus, the left lingual gyrus, and the left postcentral gyrus than the other groups (Figure 3). The IGDdep- group had stronger sgACC FC with the left dorsolateral prefrontal cortex (dlPFC) than the other groups.

**Table 2:** Whole-brain seed-based functional connectivity (FC) analysis.

**Region**	**Side**	**k_E_**	**Z**	**X**	**y**	**z**	***Post hoc* test**
**SEED: DORSAL ACC**							
Precuneus	Left	256	4.50	−2	−46	48	IGD_de+_ > IGD_de−_> Controls
Cerebellar lobule IX	Right	129	4.12	10	−42	−40	IGD_de+_ > IGD_de−_, Controls
**SEED: PREGENUAL ACC**							
Supplementary motor area	Right	352	5.11	32	6	64	IGD_de−_, Controls > IGD_de+_
Dorsomedial prefrontal cortex	Right	111	4.71	10	52	34	IGD_de−_, Controls > IGD_de+_
Precuneus	Right	184	4.46	16	−42	54	IGD_de+_, IGD_de−_> Controls
Posterior cingulate cortex	Left	359	4.02	−12	−22	42	IGD_de+_, IGD_de−_> Controls
Inferior frontal gyrus	Left	135	4.29	−42	2	16	IGD_de−_> IGD_de+_ > Controls
**SEED: SUBGENUAL ACC**							
Dorsolateral prefrontal cortex	Left	254	4.34	−36	34	38	IGD_de−_> IGD_de+_, Controls
Lingual gyrus	Left	145	4.21	−18	−86	−12	IGD_de−_, Controls > IGD_de+_
Precuneus	Left	100	3.75	−8	−62	46	Controls > IGD_de+_
Postcentral gyrus	Left	186	3.75	−42	−12	38	IGD_de−_> IGD_de+_

*Brain regions in which FC showed significant differences between groups [height threshold of uncorrected p-value < 0.001, extent threshold of contiguous k_e_ > 100 voxels (18)]*.

*IGD_dep_, Internet Gaming disorder subjects without comorbid depression; IGD_dep+_, Internet Gaming Disorder subjects with comorbid depression; ACC, anterior cingulate cortex*.

**Figure 4:**
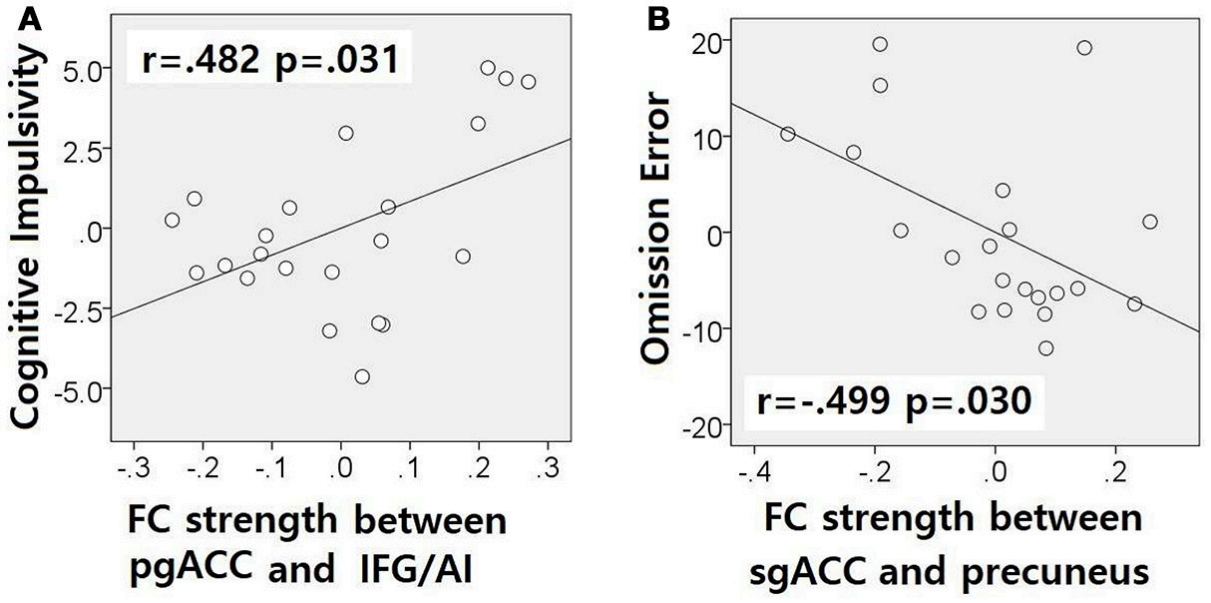
Partial correlation analyses after controlling for BDI and BAI. Non-standardized residuals were used to make scatter plots. **(A)** IGD subjects without comorbid depression exhibited a positive correlation between pgACC-IFG/AI connectivity and the BIS-cognitive impulsivity subscale score (*r* = 0.482, *p* = 0.031). **(B)** IGD subjects with comorbid depression exhibited a negative correlation between sgACC-precuneus connectivity and omission error rate in the divided attention task (*r* = −0.499, *p* = 0.030).

The correlation analysis showed a correlation between pgACC-IFG/AI connectivity strength and cognitive impulsivity in the IGDdep- group (*r* = 0.482, *p* = 0.031; Figure 4A) and a correlation between sgACC-precuneus connectivity strength and omission error in the sustained attention task in the IGDdep+ group (*r* = −0.499, *p* = 0.030; Figure 4B). The other correlation tests showed no statistical significance.

## Discussion

In this study, ACC-based FC in IGD subjects with and without depression was analyzed. Both IGD groups had stronger pgACC FC with the right precuneus, the PCC, and the left IFG/AI than the control subjects, but there were differences in FC patterns between IGD subjects with and without depression. IGD subjects with comorbid depression had stronger dACC FC with the precuneus and right cerebellar lobule IX than the other subjects. IGD subjects with comorbid depression also had weaker pgACC FC with the right dmPFC and the right SMA and weaker sgACC FC with the left precuneus, the left lingual gyrus, and the left postcentral gyrus than the other subjects. These FC alterations, which differ partially based on the presence or absence of comorbid depression, are consistent with our hypothesis that IGD patients with comorbid depression may have a characteristic neurobiological basis that contributes to their distinctive clinical features.

In comparison with other groups, IGD subjects with comorbid depression showed stronger dACC FC with the precuneus and the right cerebellar lobule IX, which have been associated with the DMN (46, 47). These findings are consistent with previous evidence that IGD subjects with comorbid depression may have hyperconnectivity between the ACC and the DMN-related brain regions, which reflects their difficulty in suppressing the DMN (20). However, the sgACC-based FC analysis showed that FC between the sgACC and the left precuneus was significantly weaker in IGD subjects with comorbid depression than in the other groups. Previous studies have indicated that the anterior and posterior DMN has asynchronous activity patterns in the depressive state (48). Our finding of weak sgACC-precuneus FC support a previous study that demonstrated changes in FC between the anterior and posterior DMN in depression (49). In addition, weak sgACC-precuneus connectivity correlated with a high omission error rate in the sustained attention task in IGD subjects with comorbid depression. A higher frequency of omission errors in IGD subjects with comorbid depression suggests that attentional problems are more pronounced in subjects with IGD when depression is involved. The significant correlation between sgACC-precuneus connectivity and omission error rate supports the hypothesis that FC alterations of the DMN contribute to impairments in attentional processes.

In comparison with the other groups, IGD subjects with comorbid depression showed weaker pgACC FC with the right dmPFC and the right SMA. It has been shown that the dmPFC is innervated by dopamine and associated with modulation of the salient and motivational values of stimuli (50). The dmPFC has been associated with reappraisal of emotional stimuli (51), and alteration of FC of the dmPFC with other brain regions has been reported in depressed patients (52, 53). The dmPFC has also been suggested to play an important role in the neurocircuitry of addiction (54). Taken together, altered FC of the dmPFC may be a crucial link between addictive Internet game use and depression. Furthermore, previous studies have shown that FC between the pgACC and the dmPFC associates closely with responses to transcranial magnetic stimulation (TMS) treatment (55) and that bupropion increases resting state FC in the dmPFC (56). Altered FC of the dmPFC has significant potential as a target of therapeutic intervention for IGD patients with comorbid depression. In addition, the SMA has been associated with cognitive control of behavior (57), and structural or functional alteration of the SMA in IGD has been reported (58, 59). Our finding of altered FC in the SMA may relate to diminished behavioral control over excessive gaming.

In comparison with controls, IGD subjects showed stronger FC between the pgACC and the left IFG/AI. Furthermore, IGD subjects without comorbid depression showed stronger pgACC-IFG/AI connectivity, which correlated significantly with higher cognitive impulsivity reflecting decision-making tendencies based on short-term satisfaction (60). Because the left IFG/AI is a seed region of the SN (61), these findings are consistent with our expectation that subjects with IGD would have increased FC of the rACC with seeds of the SN. Altered interaction between the SN and other brain networks has been suggested to contribute to the motivational, affective, and cognitive characteristics observed in addiction (62). Our current results and previous evidence (63) indicate that FC alterations in the SN, especially hyperconnectivity between the DMN and the SN, play pivotal roles in the pathophysiology of IGD. IGD subjects without comorbid depression also showed stronger sgACC FC with the left dlPFC than the other groups. Aberrant functional interactions between brain networks have been proposed as part of the pathophysiology of IGD (64, 65). Hyperconnectivity between the DMN and the central executive network may also be a neurobiological factor underlying IGD.

There were several limitations in this study. First, this study was cross-sectional, and although this study investigated the comorbidity of depression and IGD, there is currently no information about the causal relationship between the two diseases. Further longitudinal studies are needed to properly interpret the current imaging findings. Second, this study involved a small number of subjects and only focused on some of the regions of the brain even though the relationship between IGD and depression likely involves complex neurobiological mechanisms. It would be helpful to explore brain connectivity in a large number of subjects without focusing on specific seed regions of interest. Third, the study was carried out with only male subjects. Previous studies have shown that IGD is becoming more common in females (66). For the results of this study to become more generalized, further studies should include female and male gaming addicts. Finally, the study did not sufficiently control for variables that may affect the relationship between depression and IGD, and this study did not fully elucidate the brain-behavior relationship in IGD. Further studies would require broader consideration of the subjects' clinical characteristics, which may relate to their uncontrolled Internet gaming.

In conclusion, depressed and non-depressed IGD patients differed in their ACC-based FC patterns. IGD subjects with comorbid depression showed specific FC alterations in the DMN. Altered FC between the anterior and posterior DMN may associate with impaired attentional processes in IGD subjects with comorbid depression. IGD subjects with comorbid depression also had weak FC between the ACC and the dmPFC reflecting impaired regulation of emotional stimuli. Our resting fMRI results suggest that there is a neurobiological basis for the strong association between IGD and depression, which may be an important therapeutic target in the future.

## Ethics statement

All of the procedures involving human participants were performed in accordance with the ethical standards of the institutional and national research committees and with the 1964 Helsinki declaration and its later amendments. The experimental protocol was approved by the Institutional Review Board at Severance Hospital, Yonsei University, Seoul, Korea.

## Author contributions

DL and Y-CJ conceived and designed the study. JL recruited participants and acquired the imaging data. DL drafted the manuscript. KN and Y-CJ critically reviewed the manuscript and provided important intellectual content. All of the authors critically reviewed and approved the final version of this manuscript for publication.

### Conflict of interest statement

The authors declare that the research was conducted in the absence of any commercial or financial relationships that could be construed as a potential conflict of interest.
